# Kill Rate and Evaluation of *Ex Vivo* PK/PD Integration of Cefquinome Against *Actinobacillus pleuropneumoniae*

**DOI:** 10.3389/fvets.2021.751957

**Published:** 2021-12-13

**Authors:** Longfei Zhang, Hongbing Xie, Hongjuan Wang, Huanzhong Ding, Gaiping Zhang, Jianhe Hu

**Affiliations:** ^1^Postdoctoral Research Base, Henan Institute of Science and Technology, Xinxiang, China; ^2^College of Animal Science and Veterinary Medicine, Henan Institute of Science and Technology, Xinxiang, China; ^3^Postdoctoral Research Station, Henan Agriculture University, Zhengzhou, China; ^4^Guangdong Key Laboratory for Veterinary Drug Development and Safety Evaluation, South China Agricultural University, Guangzhou, China

**Keywords:** kill rate, *ex vivo* PK/PD integration, cefquinome, *Actinobacillus pleuropneumoniae*, tissue cage model

## Abstract

We wished to study the detailed and precise antibacterial activity of cefquinome against *Actinobacillus pleuropneumoniae* (APP) *in vitro* and *ex vivo*. We analyzed the relationships between kill rate and cefquinome concentration in broth and between pharmacokinetic/pharmacodynamic (PK/PD) parameters and antibacterial effect in serum and tissue cage fluid (TCF) of piglets. Cefquinome exhibited time-dependent antibacterial activity against APP according to the kill rate. The maximum kill rate was 0.48 log_10_ CFU/mL/h at the 0-9-h period in broth. In the *ex vivo* PK/PD study, the maximum concentration (C_max_), time to reach the maximum concentration (T_max_), terminal half-life (T_1/2β_), and area under the concentration time curve (AUC_infinity_) were 5.65 μg/ml, 0.58 h, 2.24 h, and 18.48 μg·h/ml in serum and 1.13 μg/ml, 2.60 h, 12.22 h, and 20.83 μg·h/ml in TCF, respectively. The values of area under the curve during 24 h/minimum inhibitory concentration (AUC_24h_/MIC) for bacteriostatic, bactericidal, and bacterial eradication effects were 18.94, 246.8, and 1013.23 h in serum and 4.20, 65.81, and 391.35 h in TCF, respectively. Our findings will provide a valuable basis for optimization of dosage regimens when applying cefquinome to treat APP infection.

## Introduction

Porcine contagious pleuropneumonia (PCP), caused by *Actinobacillus pleuropneumoniae* (APP), is a serious respiratory system diseases in pigs (1, 2). The classical symptoms of acute PCP are hemorrhagic necrotizing pneumonia and fibrinous pneumonia with high morbidity and mortality (3, 4). Long-term infected pigs would show weight decline, low feed conversion rate, and delayed sale time. Therefore, PCP has caused considerable economic losses to farmers and severely restricted the development of the pig industry. However, because the serotype of *A. pleuropneumoniae* was more than those of 19 species and there was no effectively and commonly used vaccine to protect pigs at present (5–7). Hence, antimicrobial therapy is still a rapid and efficacious method for PCP treatment, such as cephalosporins, fluoroquinolones, and macrolides (8–14). However, with the abundant unreasonable application of drugs, multidrug-resistant pathogens were gradually selected and spread all over the world. Cephalosporins had been broadly applied in veterinary and human clinical therapy, and the resistant gene can spread from animal bacteria to human bacteria. Therefore, the resistance of veterinary bacteria to cephalosporins may not only reduce the antibacterial effect but also seriously threaten the health and life of humans (15, 16). One of the major resistant mechanisms is the production of β-lactamase under selective pressure of antibiotics applied in irrational application. Therefore, a rational dosage regimen is an important method to prevent the emergence and spread of resistant bacteria. In view of these points, dosage regimens should be optimized not only to reach a clinical efficacy but also to prevent the emergence and spread of resistant bacteria.

The pharmacokinetic/pharmacodynamic (PK/PD) integration model can comprehensively study the relationship between PK/PD parameters and antibacterial effect and has been widely applied to design rational dosage regimens in clinical trials. At present, the common PK/PD model can classify to *in vitro, in vivo*, and *ex vivo* models. Each model has its own advantage. Compared to *in vitro* and *in vivo* models, the *ex vivo* model needs few animals, low cost, and less harm, and the results can partially reflect the influence of the host. The tissue cage (TC) model was an ideal method for *ex vivo* PK/PD integration and has been applied in several animals to study the *ex vivo* PK/PD for respiratory pathogens, such as *Pasteurella multocida, Mannheimia haemolytica*, and *A. pleuropneumoniae* (17–19).

Cefquinome is a broad-spectrum fourth-generation cephalosporin used only in animals. It has been approved for the treatment of several diseases caused by *A. pleuropneumoniae, Klebsiella pneumoniae*, and *Streptococcus suis* (8, 20, 21). One report (22) has applied the TC model to study the *ex vivo* PK/PD integration of cefquinome against *Escherichia coli*. As far as we know, the *ex vivo* PK/PD integration of cefquinome against APP has not been reported. Therefore, in this study, we will apply a piglet TC model to study the *ex vivo* PK/PD integration of cefquinome against APP. Meanwhile, a kill rate-based time-kill curve will be conducted to study the relationships between cefquinome concentrations and kill rates *in vitro*. We wished that these findings could precisely clarify the antibacterial activities of cefquinome against APP to design a rational dosage regimen.

## Materials and Methods

### Bacterial Strain, Drugs, and Chemicals

APP (CVCC259) was provided by the Chinese Veterinary Culture Collection Center (Qingdao, China). Standard solutions of cefquinome were purchased from the China Institute of Veterinary Drugs Control (Beijing, China). Cefquinome sulfate injection (Cobactan^®^ batch number: A673A01) was bought from Intervet International (Boxmeer, the Netherlands). Mueller–Hinton agar (MHA) and tryptic soy broth (TSB) were purchased from Guangdong Huankai Microbial Technology (Guangzhou, China). Nicotinamide adenine dinucleotide (NAD; lot: 20160810) was obtained from MYM Biological Technology (Beijing, China). Newborn bovine serum was supplied by Guangzhou Ruite Biotechnology (Guangzhou, China).

### Determination of MIC and Time-Kill Curves of Cefquinome Against APP in TSB

APP was incubated in TSB and MHA with 4% newborn bovine serum and 1% NAD (1 mg/ml). The MIC of cefquinome against APP was tested by microdilution according to the reference methods of the Clinical and Laboratory Standards Institute (23). Briefly, after the APP was incubated for 8 h in a constant temperature shaker (200 rpm, 37°C), and the exponential phase bacterium suspension was diluted to a final concentration of 5 × 10^5^ colony-forming units (CFU)/mL by 10-fold dilution. The cefquinome suspension ranging from 0.0078 to 4 mg/l was prepared after serial twofold dilutions with TSB, and 100 μl was added to a 96-well plate. After 100 μl bacterial sample was added to each well, the plate was cultured for 18–20 h in a humidified incubator (37°C, 5% CO_2_). The MIC was determined as the minimal concentration of cefquinome without visible turbidity. All experiments were repeated thrice.

For time-kill curves, eight twofold increased drug concentrations based on MIC (0.5, 1, 2, 4, 8, 16, 32, and 64 × MIC) and control group (0 × MIC) were applied in the following studies. Briefly, a series of cefquinome solutions (0, 0.39, 0.78, 1.56, 3.12, 6.25, 12.5, 25, 50 μg/ml) were diluted by sterile physiologic saline. Then, 100 μl cefquinome solution, 1 ml logarithmic phase APP (~10^7^ CFU/ml), and 8.9 ml preheated TSB were added into a 15-ml sterile tube. After mixing, the tubes were placed in an incubator (37°C, 5% CO_2_) for 24 h. During the incubation period, 100 μl of the suspension was removed from the tube at 0, 1, 3, 6, 9, 12, and 24 h, respectively. After a series of 10-fold dilution (10^−1^-10^−6^) by 0.9% NaCl, the dilutions were dropped on MHA and incubated for 18–20 h for bacterial counting. The detected limitation of the bacterial population was 50 CFU/ml. When the number of APP < 50 CFU/ml, 50 CFU/ml was used to draw kill curves. All experiments were repeated thrice. For depicting kill curves, the logarithmic mean value (Log_10_ CFU/mL) was taken as the vertical axis and the culture time was taken as the horizontal axis.

### Calculation and Integration of Kill Rate and Concentration

The antibacterial effect was described as the maximum change of APP number (Log_10_ CFU/mL) over each time period. The kill rate was the antibacterial effect divided by the corresponding time period. For kill curves, the kill rate was represented by the slope of the curve at a given time period (0–1, 1–3, 3–6, 6–9, 0–3, 0–6, and 0–9 h). Seven kill rates were obtained from different cefquinome concentrations. Finally, the kill rate (in the same period) and cefquinome concentration relationship were analyzed by using the sigmoid E_max_ model (WinNonlin 5.2.1, Pharsight, MO, USA). The equation was described as follows:


$$\begin{array}{l} E=E_{0}+\frac{\left(E_{\max}-E_{0}\right)\times C_{e}^{N}}{C_{e}^{N}+EC_{50}^{N}} \end{array}$$

where E is the kill rate; E_max_ is the maximum kill rate in TSB with cefquinome during each period; E_0_ is the kill rate in control TSB; C_e_ is the cefquinome concentration; N is the Hill coefficient that describes the steepness of the curve for the kill rate and cefquinome concentration; and EC_50_ is the cefquinome concentration producing 50% of the maximum kill rate. The coefficient of determination (R^2^) represented the relationship between kill rate and cefquinome concentration and can be acquired after sigmoid E_max_ simulation. The higher the value of R^2^, the higher the level of correlation between kill rate and concentration.

### Animals and TC Implantation

Six healthy piglets (Duroc × Landrace × Yorkshire, three females and three males, ~25 kg) were purchased from the Guangzhou Fine Breed Swine Farm (Guangzhou, China). They were housed in separate cages and fed antibiotic-free fodder twice daily, while water was provided *ad libitum*.

TCs were the same as those employed in previous study (22). The brief operation to implant TC was as follows. Firstly, a deep general anesthesia was induced by application of pentobarbital sodium into the neck by intramuscular injection. Then, the local infiltration anesthesia was induced by subcutaneous injection of procainamide hydrochloride. Thirdly, two TCs (sterilized by 75% ethyl alcohol and ultraviolet light) were implanted subcutaneously to each side of the neck equidistant from the jugular vein and spinal cord in each piglet. At last, penicillin (1000,000 IU/kg) and tetracycline ointment were used twice daily for 3 days to prevent infections. After 4–5 weeks, the TC was filled with TCF. After bacteriological examination, the TC without bacteria was used for the following experiment. The experimental protocol was approved (2016016) by the Committee on the Ethical Use of Animals of South China Agricultural University (Guangzhou, China).

### PK of Cefquinome and *Ex vivo* Antimicrobial Activity

Each piglet received cefquinome (2 mg/kg body weight) by intragluteal injection. Then, 1 ml TCF samples was collected at 0, 0.25, 0.5, 1, 3, 6, 9, 12, 24, 48, 72, and 96 h, and 5 ml blood samples was collected at 0, 0.083, 0.25, 0.5, 1, 2, 4, 6, 8, 12, 24, 36, and 48 h into sterilized centrifuge tubes, respectively. After being centrifuged (3,000 × *g*, 10 min, 4°C), the supernatant of TCF and serum samples were divided into two sterilized tubes and stored at −20°C within 2 weeks for measurement of the cefquinome concentration and *ex vivo* antimicrobial activity.

Determination of the cefquinome concentration was undertaken by high-performance liquid chromatography tandem mass spectrometry, as described in our previous study (8). PK data were analyzed based on the non-compartment model by WinNonlin 5.2.1.

The MIC test methods for serum and TCF samples were the same as in the TSB sample. For *ex vivo* kill curves, 5 μl exponential phase bacterial suspension (~10^9^ CFU/ml) was added to sterilized tubes containing 0.5 ml TCF samples (for serum, 10 μl suspension was added to 1 ml serum sample) to ensure that the final bacterial concentration was about 5 × 10^7^ CFU/ml and cultured in a humidified incubator (37°C, 5% CO_2_). Then, 50 μl suspension sample was removed at 0, 1, 3, 6, 9, 12, and 24 h for APP counting. The *ex vivo* antibacterial time-kill curves were depicted the same as described in TSB. The antibacterial effect was described as the maximum change of APP number (Log_10_ CFU/mL) over 24 h.

### *Ex vivo* PK/PD Integration

For PK/PD integration, the AUC_24h_/MIC (AUC_24h_ was the cefquinome concentration of each sample multiplied by 24 h) was applied to evaluate the *ex vivo* antibacterial effect by the sigmoid E_max_ model. The model equation was described as follows:


$$\begin{array}{l} I=I_{0}+\frac{\left(I_{0}-I_{\max}\right)\times C_{e}^{N}}{C_{e}^{N}+IC_{50}^{N}}\; \end{array}$$

where I is the antibacterial effect; I_0_ is the maximum change in Log_10_ CFU/mL of the control sample (drug free) after incubation for 24 h; I_max_ is the maximum reduction of bacterial count (Log_10_ CFU/mL) in TCF or serum samples (contain cefquinome) after incubation for 24 h; C_e_ is the value of AUC_24h_/MIC; IC_50_ is the value of AUC_24h_/MIC needed to reach 50% I_max_; and N is the Hill coefficient that describes the steepness of the curve for AUC_24h_/MIC and antibacterial effect.

The antibacterial effect of cefquinome was quantified for three levels according to the value of I. I = 0 was defined as a “bacteriostatic” effect. I = −3 was considered a “bactericidal” effect. I = −4 was defined as a “bacterial-eradication” effect.

### Statistical Analysis

The one-way ANOVA test (SPSS, version 22, IBM) was applied to analyze the difference of cefquinome PK indices among sera and TCF of each piglet. *p* < 0.05 indicated significant difference.

## Results

### MIC and Kill Curves of Cefquinome Against APP in TSB

**Figure 1:**
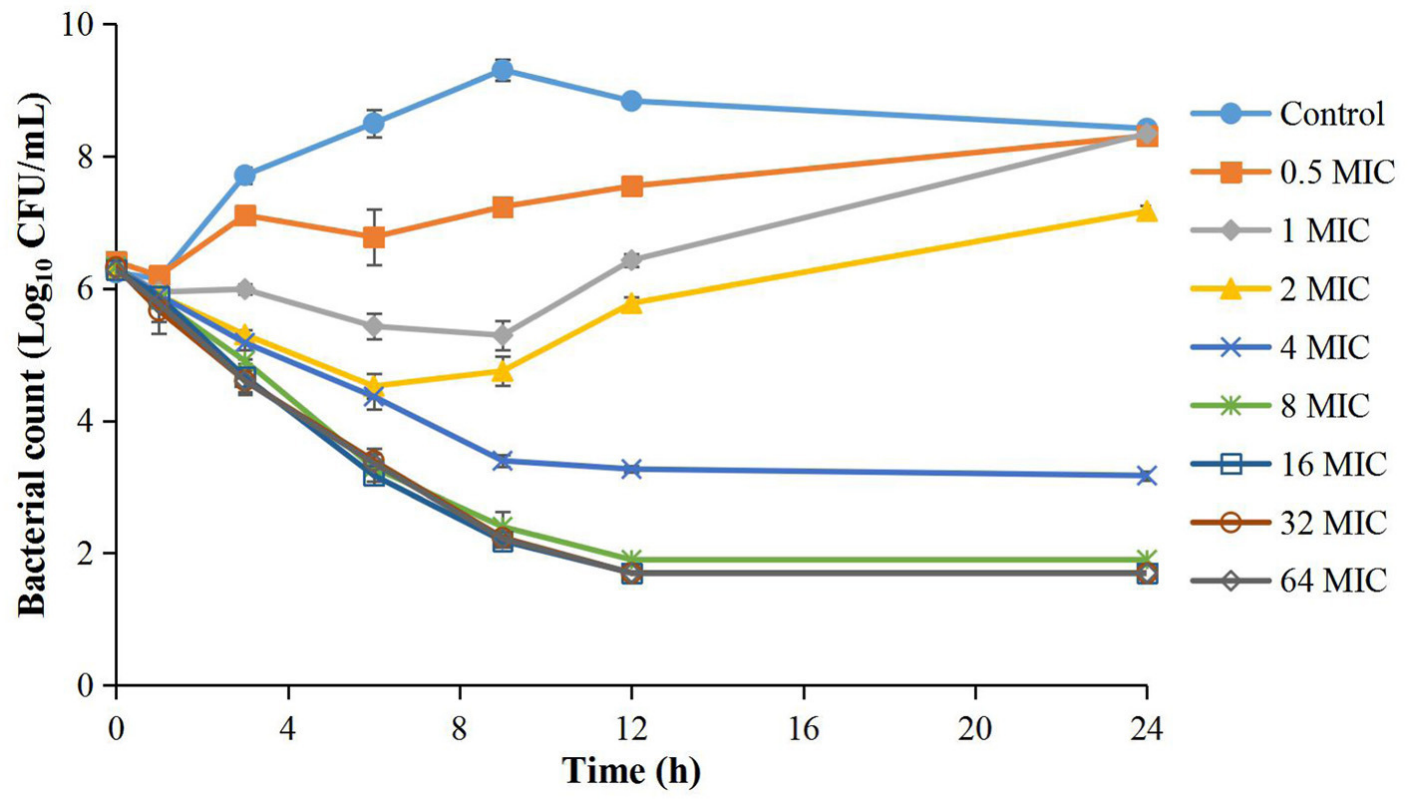
Time-kill curves of different concentrations of cefquinome against *A. pleuropneumoniae* in TSB. Each symbol represents the mean ± SD after the experiment had been repeated thrice.

The MIC of cefquinome against APP was 0.008 μg/ml in TSB. The time-kill curves are depicted in Figure 1. The reduction of the APP population ranged from 1.02 to 6.32 Log_10_ CFU/mL at different cefquinome concentrations (1 to 64 × MIC) during 0 to 24 h. At 0.5 × MIC, a slight increase (1.91 Log_10_ CFU/mL) in APP number was observed. At 1 × MIC and 2 × MIC, the total reduction of APP was 1.02 Log_10_ CFU/mL and 1.08 Log_10_ CFU/mL, respectively, and a regrowth of APP was observed after 9 h. At 4 × MIC, a bactericidal effect (−3.16 Log_10_ CFU/mL) was achieved without regrowth. At 8 × MIC, a bacterial-eradication effect (−4.43 Log_10_ CFU/mL) was reached. At 16 × MIC, the antibacterial effect reached a maximum value (−6.29 Log_10_ CFU/mL) and no longer increased with drug concentration added.

### Integration of the Kill Rate and Cefquinome Concentration

Because the APP population was lower than the limitation of detection after 9 h during 16 to 64 × MIC, we only calculated and analyzed the kill rate during 0 to 9 h for precise PK/PD integration (Table 1). At each time period, the kill rate increased upon addition of cefquinome during low drug concentrations (0–8 × MIC) and did not obviously increase and showed no significant difference during high drug concentrations (8–64 × MIC). At each drug concentration, the kill rate decreased gradually along with the prolongation of the incubation time. Therefore, a long interaction time was needed to produce bactericidal effect (6 h for 8 × MIC, 12 h for 4 × MIC).

**Table 1:** Kill rate (Log_10_ CFU/mL/h) at different time intervals at different concentrations of cefquinome against *A. pleuropneumoniae* in TSB.

	**Concentrations of cefquinome in TSB (× ** **MIC)**							
**Time (h)**	**0**	**0.5**	**1**	**2**	**4**	**8**	**16**	**32**	**64**
0–1	0.09	0.21	0.36	0.42	0.43	0.51	0.40	0.65	0.51
1–3	−0.70	−0.46	−0.02	0.30	0.36	0.46	0.61	0.53	0.59
3–6	−0.26	0.11	0.19	0.26	0.27	0.54	0.50	0.40	0.41
6–9	−0.27	−0.15	0.05	−0.08	0.32	0.30	0.33	0.39	0.38
0–3	−0.49	−0.23	0.11	0.34	0.38	0.48	0.54	0.57	0.56
0–6	−0.50	−0.06	0.15	0.30	0.33	0.51	0.52	0.49	0.49
0–9	−0.34	−0.09	0.11	0.17	0.33	0.44	0.46	0.45	0.45

*Values are the mean of experiments repeated thrice*.

**Figure 2:**
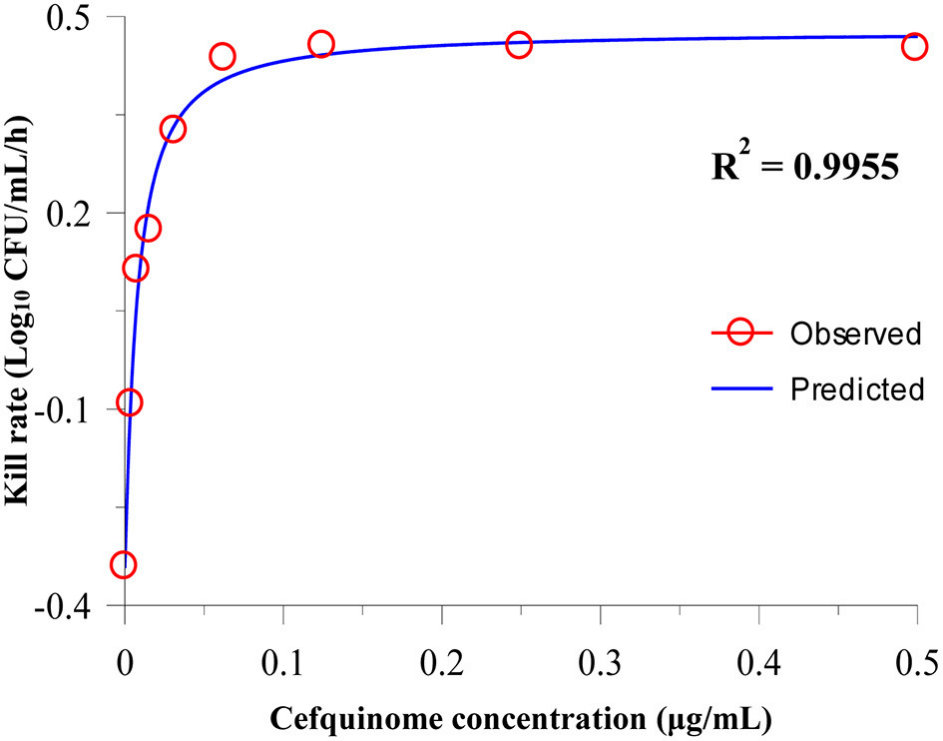
Relationship between the cefquinome concentration and kill rate during 0 h to 9 by Sigmoid E_max_ simulation. The R^2^ value is the coefficient of determination.

After analyzing the relationship between cefquinome concentration and kill rate during 0 to 9 h by the sigmoid E_max_ model, the main parameters and correlations (R^2^) are as listed in Table 2. R^2^ ranged from 0.9183 to 0.9955. The fittest relationship was at the 0-9-h time period (R^2^ = 0.9955) (Figure 2). The maximum kill rate was 0.48 Log_10_ CFU/mL/h. The kill rate increased rapidly with increasing drug concentration from 0.5 × MIC to 8 × MIC and then increased slowly from 8 × MIC to 16 × MIC (Figure 2). The kill rate was stable and virtually unchanged when the cefquinome concentration >16 × MIC.

**Table 2:** Main parameters between the cefquinome concentration and kill rate after sigmoid E_max_ simulation.

**Time**	**E_**max**_**	**EC_**50**_**	**E_**0**_**	**N**	**R^**2**^**
**(h)**	**(Log_**10**_ CFU/mL/h)**	**(μg/mL)**	**(Log_**10**_ CFU/mL/h)**		
0–1	0.5408	0.0071	0.0895	0.9883	0.9183
1–3	0.5480	0.0075	−0.7198	1.7084	0.9937
3–6	0.4743	0.0045	−0.2585	0.8631	0.9627
6–9	0.3990	0.0145	−0.2588	1.1948	0.9529
0–3	0.5424	0.0069	−0.5018	1.5435	0.9952
0–6	0.5157	0.0049	−0.4982	1.0821	0.9949
0–9	0.4777	0.0077	−0.3405	1.1043	0.9955

*E_max_, the maximum kill rate during each period; EC_50_, the cefquinome concentration producing 50% of the maximum kill rate; E_0_, the kill rate in control TSB; N, the Hill coefficient that describes the steepness of the curve for the kill rate and cefquinome concentration; R^2^, the correlation coefficient between the kill rate and cefquinome concentration*.

### PK of Cefquinome and *Ex vivo* Antibacterial Activity in Serum and TCF

**Figure 3:**
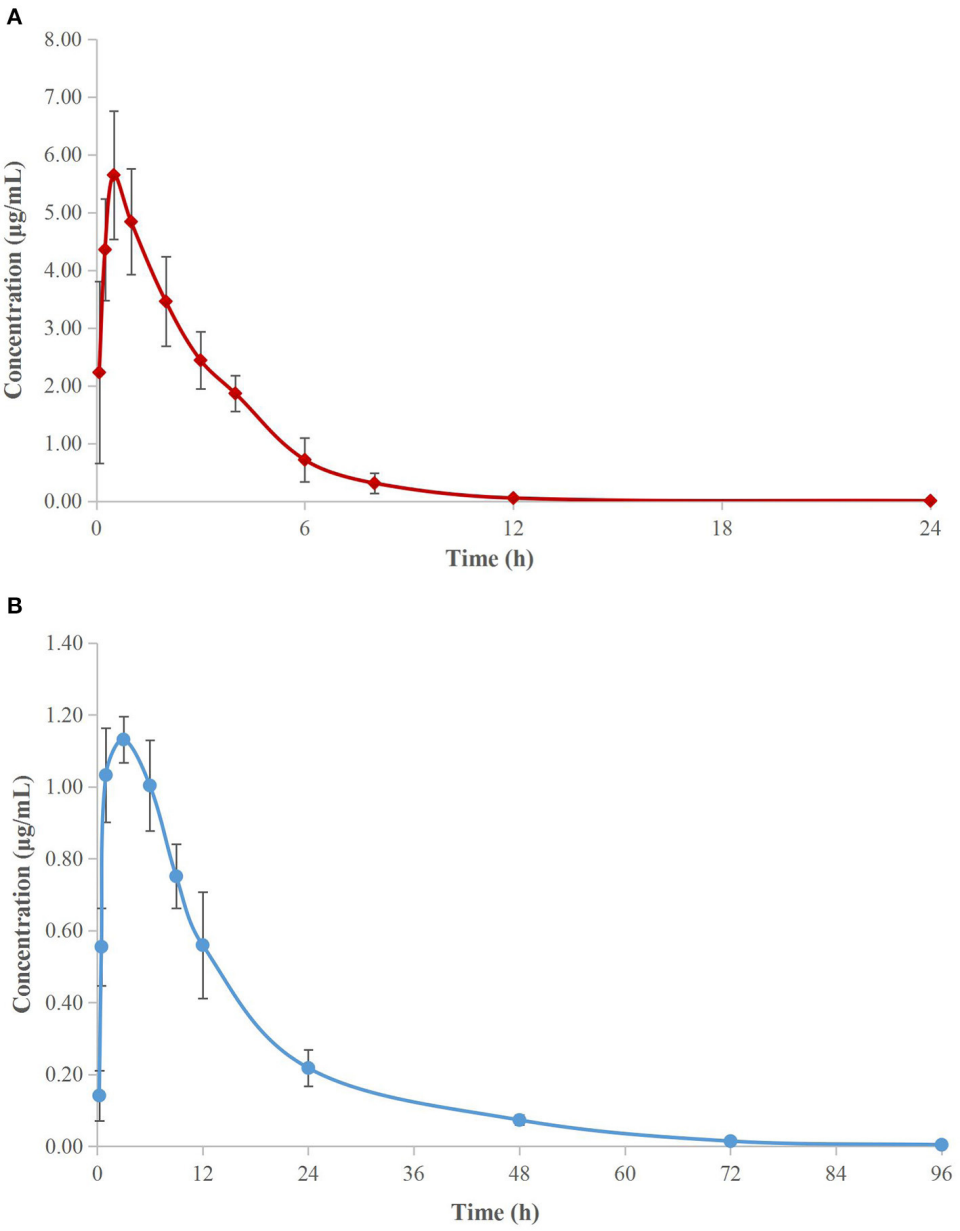
Semi-logarithmic concentration-time curves of cefquinome in porcine serum **(A)** and TCF **(B)**. Values are the mean ± standard deviation (*n* = 6).

The concentration–time curves of cefquinome are depicted in Figure 3A (serum) and Figure 3B (TCF). The main PK parameters are listed in Table 3. The mean values of the maximum concentration (C_max_) were 5.65 ± 1.10 μg/ml and 1.13 ± 0.06 μg/ml, the time needed to reach the maximum concentration (T_max_) were 0.58 ± 0.20 h and 2.6 ± 0.8 h, the area under the concentration–time curves (AUC_infinity_) were 18.48 ± 3.28 μg h/ml and 20.83 ± 0.57 μg h/ml, and the terminal half-lives (T_1/2β_) were 2.24 ± 0.13 h and 12.22 ± 0.85 h in serum and TCF, respectively.

**Table 3:** Pharmacokinetics of cefquinome in serum and TCF after intramuscular administration at 2 mg/kg body weight.

**Variable (units)**	**Serum**	**TCF (p)**
T_*max*_ (h)	0.58 ± 0.20	2.60 ± 0.8 (0.00)
C_*max*_ (μg/mL)	5.65 ± 1.10	1.13 ± 0.06 (0.00)
T_1/2β_ (h)	2.24 ± 0.13	12.22 ± 0.85 (0.00)
AUC_*last*_ (μg∙h/mL)	18.47 ± 3.28	19.49 ± 2.87 (0.56)
AUC_*infinity*_ (μg∙h/mL)	18.48 ± 3.28	20.82 ± 0.57 (0.47)
MRT_*last*_ (h)	2.80 ± 0.36	16.85 ± 0.61 (0.00)
Cl/F (L/kg)	0.14 ± 0.02	0.096 ± 0.003 (0.01)
V_d_/F (L/kg)	0.45 ± 0.07	1.92 ± 0.49 (0.00)

*Values are the mean ± standard deviation (SD), n = 6. T_max_, time needed to reach the maximum concentration; C_max_, maximum concentration; T_1/2β_, terminal half-life; AUC_last_, AUC computed from time zero to the time of the last positive Y value; AUC_infinity_, AUC from time zero extrapolated to infinity; MRT_last_, mean residence time when the drug concentration is based on values up to and including the last measured concentration; F, bioavailability; V_d_/F, volume of distribution during the terminal phase divided by bioavailability; Cl/F, body clearance divided by bioavailability. p <0.05 indicated significant differences*.

**Figure 4:**
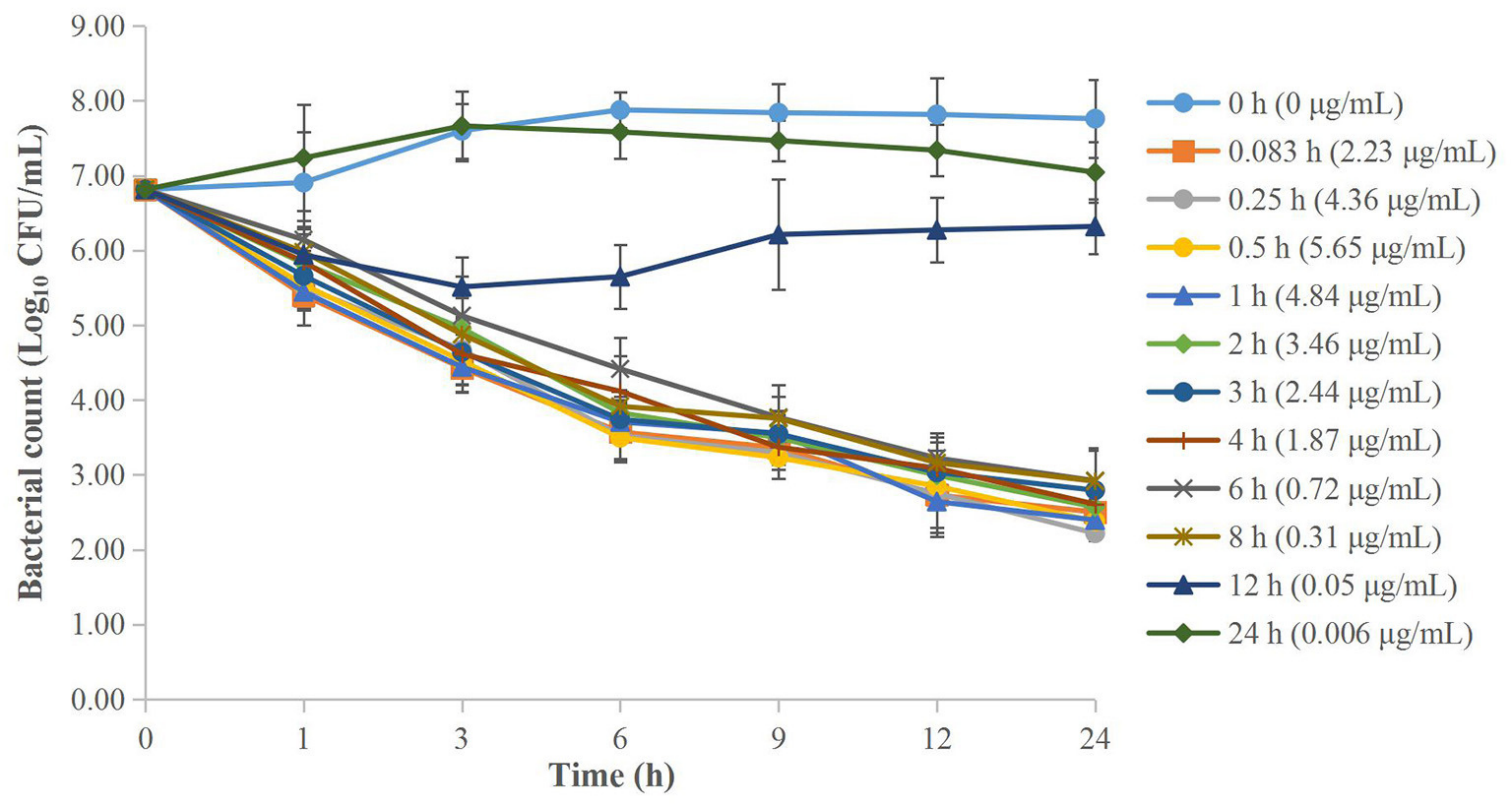
*Ex vivo* time-kill curves of cefquinome against *A. pleuropneumoniae* in serum before and after intramuscular administration. Values are the mean ± standard deviation (*n* = 6).

**Figure 5:**
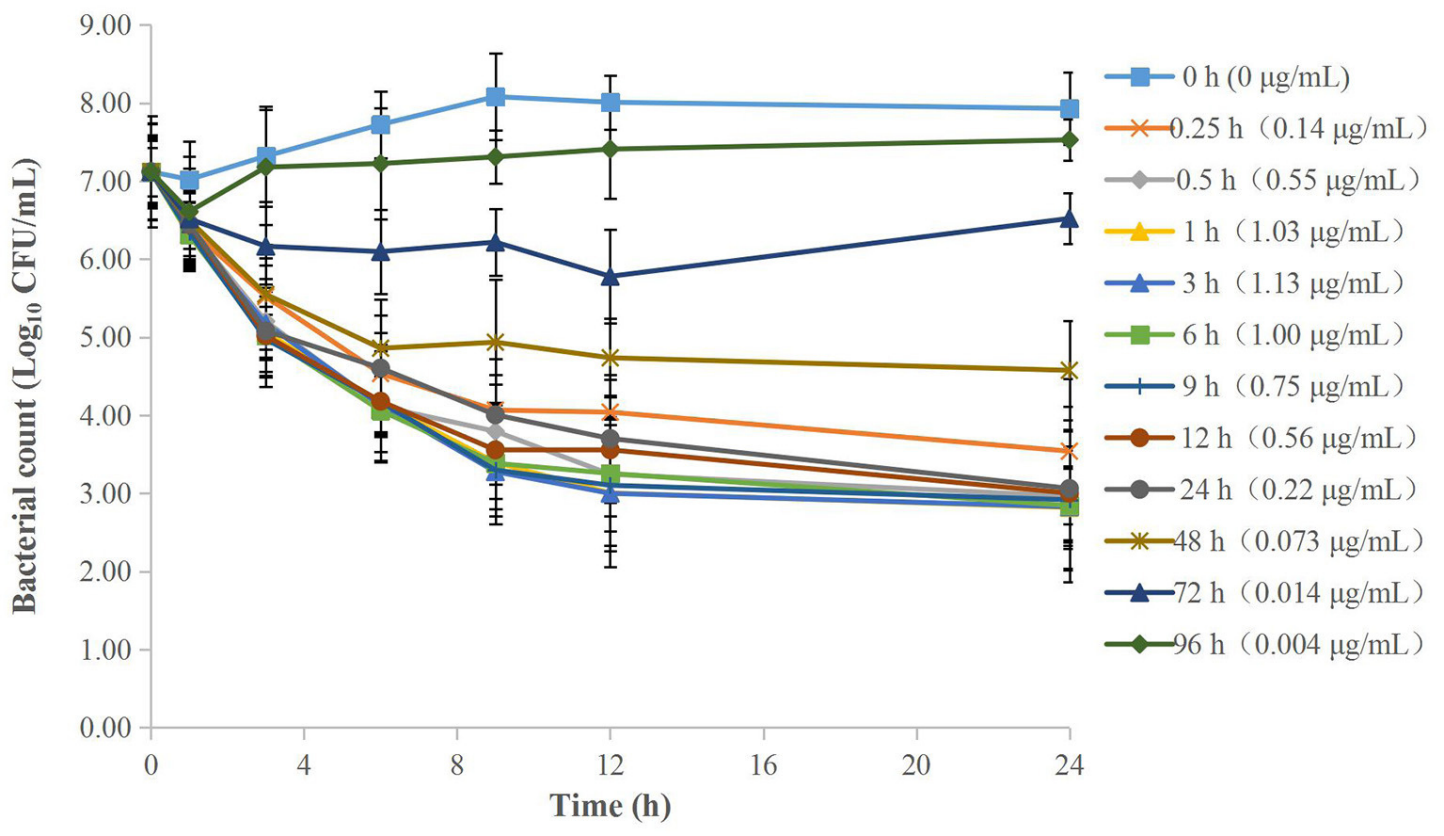
*Ex vivo* time-kill curves of cefquinome against *A. pleuropneumoniae* in TCF before and after intramuscular administration. Values are the mean ± standard deviation (*n* = 6).

The MICs of cefquinome against APP were both 0.016 μg/ml in serum and TCF. The *ex vivo* time-kill curves of cefquinome against APP in serum and TCF are depicted in Figures 4, 5 respectively. For serum samples, a bacteriostatic effect (1.30 Log_10_ CFU/ml reduction) could be achieved for samples collected at 12 h and APP was regrown after 3 h. The bactericidal effect (3.89 and 3.90 Log_10_ CFU/ml reduction) was observed for samples collected at 6 and 8 h. The bacterial-eradication effect (4.02 to 4.60 Log_10_ CFU/ml reduction) was produced for samples collected at 0.083 to 4 h. For TCF, samples collected at 0.5 to 24 h exerted a bacterial-eradication effect (4.06 to 4.30 Log_10_ CFU/ml reduction) without regrowth after 24 h of incubation. Bactericidal activity (3.58 Log_10_ CFU/ml reduction) was observed for the sample collected at 0.25 h. The bacteriostatic effect (0.15 to 2.54 Log_10_ CFU/ml reduction) was observed for samples collected at 48 to 96 h.

### *Ex vivo* PK/PD Integration and Analyses

**Figure 6:**
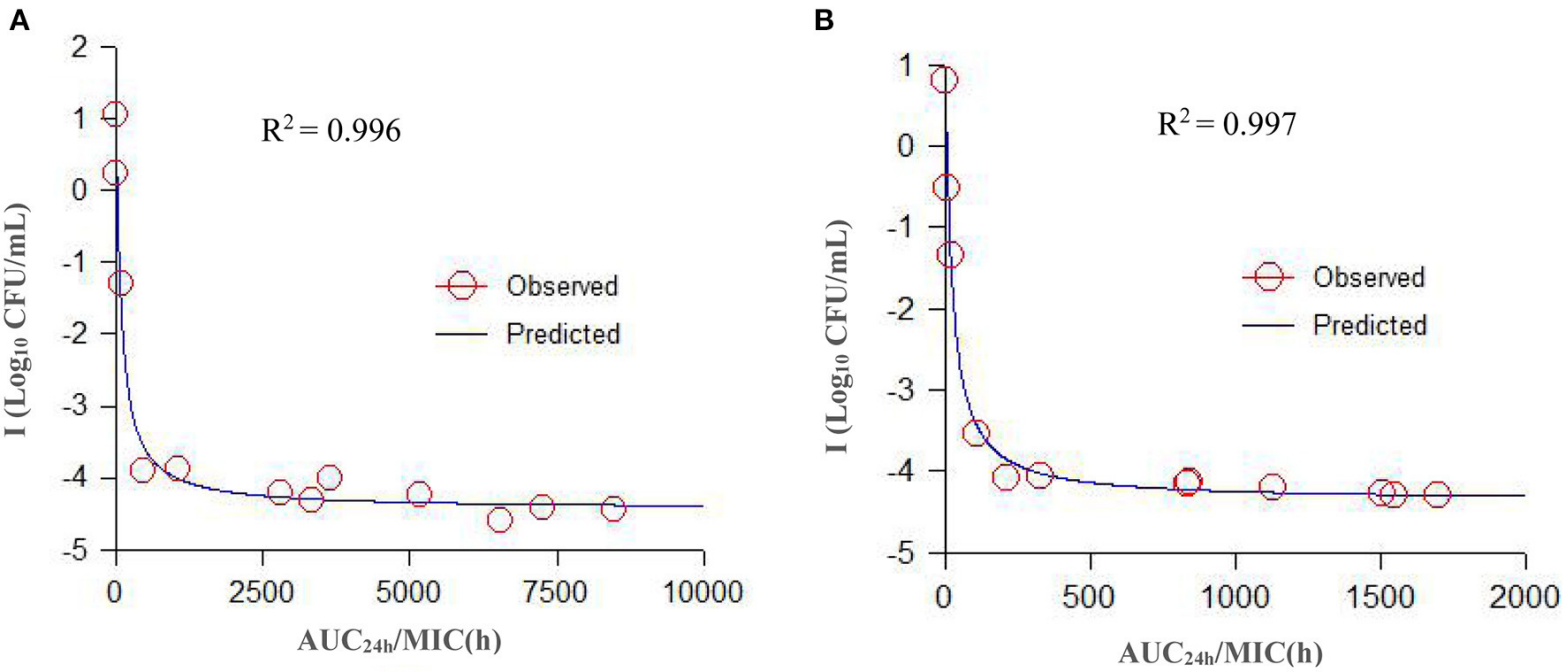
A specimen plot of *ex vivo* AUC_24h_/MIC-antibacterial effect (Log CFU/mL) for *A. pleuropneumoniae* in serum **(A)** and TCF **(B)**. The R^2^ value is the coefficient of determination. Values are the mean ± standard deviation (*n* = 6). SD bars have been omitted for clarity.

The parameters integrated by the PK/PD model are presented in Table 4. A specimen plot of AUC_24h_/MIC-effect (Log_10_ CFU/mL) is depicted in Figure 6A (serum) and Figure 6B (TCF). The values of AUC_24h_/MIC to produce a bacteriostatic effect, bactericidal effect, and bacterial-eradication effect were 18.94, 246.8, and 1013.23 h in serum, and 4.20, 65.81, and 391.35 h in TCF, respectively.

**Table 4:** Values of *ex vivo* PK/PD parameters and AUC_24h_/MIC required to achieve various degrees of antibacterial efficacy in serum and TCF.

**Parameter (units)**	**Serum**	**TCF**
I_max_ (Log_10_ CFU/mL)	−4.43	−4.36
I_0_ (Log_10_ CFU/mL)	0.93	0.72
I_max_ – I_0_ (Log_10_ CFU/mL)	−5.37	−5.08
IC_50_ (h)	90.43	24.54
Slope (N)	1.00	1.02
AUC_24h_/MIC for bacteriostatic effect (h)	18.94	4.2
AUC_24h_/MIC for bactericidal effect (h)	246.8	65.81
AUC_24h_/MIC for eradication effect (h)	1013.23	391.35

*I_max_, the maximum reduction in bacterial count in Log_10_ CFU/mL in serum or TCF with cefquinome during 24 h; I_0_, the change in bacterial count in serum or TCF with no drugs during 24 h; IC_50_, the AUC_24h_/MIC value required to achieve 50% of the maximal antibacterial effect during 24 h; N, the Hill coefficient which describes the steepness of the curve for AUC_24h_/MIC*.

## Discussion

*Actinobacillus pleuropneumoniae* is a serious pathogenic bacterium which can cause respiratory diseases of pigs (24, 25). Antibacterials were an important therapeutic method. However, with unreasonable application of a large number of drugs, multidrug-resistant pathogens gradually appeared which may reduce the antibacterial effect and threaten human health. To solve drug resistance, development of new drugs and optimization of drug dosage regimens were two effective methods. However, the development rate of new drugs cannot keep up with the emergence of drug resistance. Therefore, it is a practical method to optimize the drug schemes.

For dosage regimen design, the commonly applied PD parameter was MIC. However, because of the limitation of the test method, there are some deficiencies. One deficiency is that the MIC was tested in static drug concentrations during a period which is known to have an “all or nothing” value. Hence, the MIC reflects only the final antibacterial effect, which does not clarify the dynamic process of antibacterial activity at different periods. The other deficiency is that the MIC is commonly examined in an artificial medium, which does not consider the interaction of body substances (e.g., neutrophils, cytokines, immune factors) (26, 27). Therefore, for a detailed understanding of antibacterial activity, a kill rate-based time-kill curve has been applied for *in vitro* PK/PD integration. This method can provide more valuable data by reflecting the dynamic interaction from multidimensional aspects of drugs against pathogens. The kill rate also has been applied to classify drugs as time-dependent or concentration-dependent (28). Therefore, in the present study, we applied this model to study the relationship between concentrations and kill rate of cefquinome against APP during different time periods.

Our *in vitro* PK/PD results show that the kill rates of cefquinome against APP were relatively low. Even when the drug concentration reached 64 × MIC, the maximum kill rate was 0.59 Log_10_ CFU/mL/h. The kill rate reached a maximum value when the drug concentration reached 8 × MIC and did not increase with the drug concentration added. We also found that the antibacterial activity of cefquinome against APP was according to time-dependent drug characteristics. That is to say, a more potent antibacterial effect required the cefquinome concentration to be higher than the MIC for a longer time. These findings were similar to other cephalosporin studies. Ahmad et al. (29) applied a pharmacodynamic model to analyze the relationship between the kill rate and concentration of cefquinome against *Staphylococcus aureus*, and the maximum kill rate was 1.67 h^−1^. Thomas et al. (30) studied the antibacterial activity of cefquinome against equine bacteria. They showed that the effect of cefquinome was time-dependent, but that the kill rate and drug concentration had no obvious correlation. Maneke et al. (31) compared the kill rate of cefalexin and kanamycin alone or combination against five mastitis pathogens. The results show that the higher concentrations can produce faster kill for most strains. At lower antibiotic concentrations, a faster and greater kill was observed for the combination of cefalexin and kanamycin than alone. In our study, a sigmoid E_max_ model was applied to analyze the relationship between cefquinome concentrations and kill rate. As far as we know, one report (32) also applied this method to study the antibacterial activity of doxycycline against *Mycoplasma gallisepticum*. Their results showed that doxycycline exhibited time-dependent antibacterial activity and the maximum kill rate was 0.11 h^−1^.

The PK properties of cefquinome in piglet serum and TCF were reported (22) after intramuscular administration at 2 mg/kg. The reported C_max_, T_max_, AUC, and T_1/2β_ were 6.15 and 1.15 μg/ml, 0.34 and 3.00 h, 21.40 and 17.79 μg h/ml, and 2.30 and 11.81 h, in serum and TCF, respectively, and were similar to our values.

For PK/PD integration, the commonly applied PK/PD indices included C_max_/MIC, AUC_24h_/MIC, and %T > MIC (the percentage of time of drug concentration above MIC after each dosing interval). Generally, the fittest PK/PD parameter for time-dependent drugs was %T > MIC after different dosage treatments in dynamic models. Several reports had studied the PK/PD integration of cefquinome against other pathogens, such as *Streptococcus suis, Haemophilus parasuis, Escherichia coli*, and *Staphylococcus aureus* (21, 33–35), and they had calculated the different values of %T > MIC for achieving different antibacterial effect to guide the designation of dose regimens. However, in our present study, we applied AUC_24h_/MIC for the *ex vivo* PK/PD integration of cefquinome against APP. Because the different values of %T > MIC cannot be acquired in static drug concentrations, AUC_24h_/MIC had been widely applied for *ex vivo* PK/PD analysis in several kinds of drugs and bacteria. Aliabadi and Lees (17) used a TC model in goats to study the *ex vivo* PK/PD integration of danofloxacin against *Mannheimia haemolytica*, and the values of AUC_24h_/MIC in serum for bacteriostatic, bactericidal, and bacterial-eradication effects were 22.6, 29.6, and 52.2 h, respectively. Dorey et al. (18) applied a TC model in pigs to study the *ex vivo* PK/PD integration of oxytetracycline against APP, and the values of AUC_24h_/MIC to reach bacteriostatic, bactericidal, and bacterial-elimination effects were 33.1, 55.4, and 79.7 h, respectively. Zhou et al. (19) applied a TC model in pigs to study the relationships between *ex vivo* PK/PD parameters and the antibacterial effect of tulathromycin against *Pasteurella multocida* in serum and TCF, and the values of AUC_24h_/MIC for bacteriostatic, bactericidal, and bacterial-eradication effects were 44.55, 73.19, and 92.44 h in serum, respectively (the values of AUC_24h_/MIC were 32.42 and 41.85 for bacteriostatic and bactericidal effects in TC exudates, respectively). Zhang et al. (22) used a piglet TC model to study the *ex vivo* PK/PD integration of cefquinome against *Escherichia coli* ATCC 25922, and the values of AUC_24h_/MIC for bactericidal and bacterial-eradication effects were 35.01 and 44.28 h in TCF, respectively. Sun et al. (9) studied the *ex vivo* PK/PD integration of ceftiofur against APP in swine serum. Their results showed that ceftiofur conducted a time-dependent antibacterial activity with a partly concentration-dependent pattern against APP-BW39, and the values of AUC_24h_/MIC for bacteriostatic, bactericidal, and eradication effects were 45.73, 63.83, and 69.04 h, respectively. Luo et al. (36) studied the *ex vivo* PK/PD integration of ceftiofur against *Streptococcus suis* in pig pulmonary epithelial lining fluid, and the values of AUC_24h_/MIC for achieving bacteriostatic, bactericidal, and eradication effects were 6.54, 9.69, and 11.49 h, respectively. In our present study, the *ex vivo* time-kill curves of cefquinome against APP showed no obvious differences between serum and TCF. The values for the time above the MIC after a single administration (T > MIC) were >12 h in serum and >48 h in TCF after administration at 2 mg/kg. The values of AUC_24h_/MIC for bacteriostatic, bactericidal, and bacterial-eradication effects were 18.94, 246.8, and 1013.23 h in serum, and 4.20, 65.81, and 391.35 h in TCF, respectively.

At present, the PK/PD model can generally be classified to *in vitro, ex vivo*, and *in vivo* models. Each of these models has its own advantages and disadvantages. In the present study, we conducted *in vitro* and *ex vivo* PK/PD research. Compared to the *in vivo* study, these two studies can exhibit three different antibacterial characteristics. First, the *in vitro* study can precisely and directly reflect the antibacterial activity of drug–pathogen interactions. In addition, the *in vitro* study was more fit to identify the characteristics of drugs to time or concentration-dependent antibacterial activity than the *in vivo* model. Further, the *ex vivo* study could in detail reflect the difference of protein binding, nutrient substance, immune factors, and cytokines in body fluid (such as serum, plasma, TCF, and inflammatory exudate). For the *in vivo* study, the antibacterial effect is the final result of drug, pathogen, and host interactions which cannot describe the separately interactive process of drugs and host. Therefore, it is maybe more rational and effective to comprehensively consider the data from *in vitro, ex vivo*, and *in vivo* studies during design dosage regimens.

Although we had successfully studied the *in vitro* and *ex vivo* PK/PD integration of cefquinome against APP, there were still some limitations. One is that the TCF was collected between skin and muscle. Therefore, the substance and cefquinome PKs were different from the target organs (lung) of respiratory pathogens. The other limitation is the difference between health and infected body conditions. Actually, the immune system will be activated and produce a lot of immune factors by pathogen infection. In addition, to calculate the recommended dosage regimens, an MIC_90_ was needed. Because the number of APP was not enough, we did not calculate the recommended dosages in this study. Therefore, in our further studies, we will optimize the *ex vivo* PK/PD model from these aspects, such as a microdialysis to collect lung fluids.

## Conclusions

Cefquinome exhibited time-dependent antibacterial activity against APP based on the analyses of kill rate. The maximum kill rate was 0.48 Log_10_ CFU/ml/h at 0-9 h in TSB. The time of cefquinome concentrations exceeded MIC for up to 12 h in serum and 48 h in TCF after 2 mg/kg IM administration. The values of AUC_24h_/MIC for a bactericidal effect should reach 65.81 h in TCF and 246.8 h in serum.

## Data Availability Statement

The raw data supporting the conclusions of this article will be made available by the authors, without undue reservation.

## Ethics Statement

The animal study was reviewed and approved by Committee on the Ethics of Animals of South China Agricultural University.

## Author Contributions

LZ and HW contributed to the methodology, software use, validation, data analysis, writing, and project administration. HX, HD, GZ, and JH contributed to study supervision. LZ and HX contributed to funding acquisition. All authors contributed to the article and approved the submitted version.

## Funding

This work was supported by the Postdoctoral Research Foundation of Henan Institute of Science and Technology, Key Technology Research and Development Program of Henan Province (212102110373 and 202102110241), the Scientific Research Program of Henan Institute of Science and Technology (103010620002/004), Postdoctoral Research Foundation of Henan Province (10303015/005), and Program for Innovative Research Team (in Science and Technology) in University of Henan Province (22IRTSTHN026).

## Conflict of Interest

The authors declare that the research was conducted in the absence of any commercial or financial relationships that could be construed as a potential conflict of interest.

## Publisher's Note

All claims expressed in this article are solely those of the authors and do not necessarily represent those of their affiliated organizations, or those of the publisher, the editors and the reviewers. Any product that may be evaluated in this article, or claim that may be made by its manufacturer, is not guaranteed or endorsed by the publisher.
